# Supplementary material for: Modeling glioblastoma heterogeneity as a dynamic network of cell states
Source: Mol Syst Biol. 2021 Sep 16;17(9):e10105. doi: 10.15252/msb.202010105 (PMC8444284; doi:10.15252/msb.202010105)
Supplement: Supplementary file 6 — Source Data for Figure 5 [file MSB-17-e10105-s004.zip › Figure5A_sourcedata/GSEA_3017/nosigdb_stateE.GseaPreranked.1622795031093/RICHARDS_NOWAKOWSKI_SCIENCE_2017_MURAL_UPREG.html]

Details for gene set RICHARDS\_NOWAKOWSKI\_SCIENCE\_2017\_MURAL\_UPREG[GSEA]

|  || Dataset | state63017 |
| Phenotype | NoPhenotypeAvailable |
| Upregulated in class | na\_pos |
| GeneSet | RICHARDS\_NOWAKOWSKI\_SCIENCE\_2017\_MURAL\_UPREG |
| Enrichment Score (ES) | 0.5342968 |
| Normalized Enrichment Score (NES) | 3.8270447 |
| Nominal p-value | 0.0 |
| FDR q-value | 0.0 |
| FWER p-Value | 0.0 |
Table: GSEA Results Summary

  

Fig 1: Enrichment plot: RICHARDS\_NOWAKOWSKI\_SCIENCE\_2017\_MURAL\_UPREG      
 Profile of the Running ES Score & Positions of GeneSet Members on the Rank Ordered List

  

| PROBE | GENE SYMBOL | GENE\_TITLE | RANK IN GENE LIST | RANK METRIC SCORE | RUNNING ES | CORE ENRICHMENT || 1 | PMP22 |  |  | 0 | 1.393 | 0.0423 | Yes |
| 2 | ID3 |  |  | 6 | 0.972 | 0.0661 | Yes |
| 3 | SPARCL1 |  |  | 7 | 0.963 | 0.0954 | Yes |
| 4 | LPL |  |  | 9 | 0.958 | 0.1233 | Yes |
| 5 | ANXA1 |  |  | 12 | 0.826 | 0.1461 | Yes |
| 6 | CD44 |  |  | 14 | 0.806 | 0.1694 | Yes |
| 7 | CEBPD |  |  | 15 | 0.791 | 0.1935 | Yes |
| 8 | ANXA2 |  |  | 18 | 0.733 | 0.2134 | Yes |
| 9 | PRSS23 |  |  | 21 | 0.722 | 0.2330 | Yes |
| 10 | CNN3 |  |  | 24 | 0.702 | 0.2521 | Yes |
| 11 | IFITM3 |  |  | 26 | 0.682 | 0.2717 | Yes |
| 12 | CALD1 |  |  | 41 | 0.580 | 0.2732 | Yes |
| 13 | MEG3 |  |  | 42 | 0.562 | 0.2902 | Yes |
| 14 | PMEPA1 |  |  | 44 | 0.544 | 0.3056 | Yes |
| 15 | IFITM2 |  |  | 46 | 0.536 | 0.3207 | Yes |
| 16 | SERPINE1 |  |  | 52 | 0.526 | 0.3310 | Yes |
| 17 | F3 |  |  | 55 | 0.506 | 0.3440 | Yes |
| 18 | MRC2 |  |  | 56 | 0.505 | 0.3594 | Yes |
| 19 | WLS |  |  | 65 | 0.472 | 0.3645 | Yes |
| 20 | SLC1A3 |  |  | 66 | 0.472 | 0.3789 | Yes |
| 21 | CDH6 |  |  | 70 | 0.453 | 0.3892 | Yes |
| 22 | HSPA5 |  |  | 73 | 0.449 | 0.4005 | Yes |
| 23 | SYTL2 |  |  | 82 | 0.437 | 0.4046 | Yes |
| 24 | COL5A2 |  |  | 83 | 0.434 | 0.4178 | Yes |
| 25 | CD59 |  |  | 89 | 0.418 | 0.4247 | Yes |
| 26 | TMSB4X |  |  | 93 | 0.412 | 0.4338 | Yes |
| 27 | ATF3 |  |  | 101 | 0.397 | 0.4378 | Yes |
| 28 | GPM6B |  |  | 105 | 0.391 | 0.4462 | Yes |
| 29 | CD99 |  |  | 107 | 0.389 | 0.4569 | Yes |
| 30 | LGALS1 |  |  | 113 | 0.368 | 0.4624 | Yes |
| 31 | FNDC3B |  |  | 123 | 0.354 | 0.4628 | Yes |
| 32 | VMP1 |  |  | 127 | 0.351 | 0.4700 | Yes |
| 33 | RHOB |  |  | 148 | 0.335 | 0.4571 | Yes |
| 34 | PRDM1 |  |  | 156 | 0.327 | 0.4590 | Yes |
| 35 | ZFP36L2 |  |  | 162 | 0.324 | 0.4631 | Yes |
| 36 | SPATS2L |  |  | 163 | 0.323 | 0.4729 | Yes |
| 37 | JUN |  |  | 172 | 0.316 | 0.4734 | Yes |
| 38 | ARHGAP29 |  |  | 175 | 0.314 | 0.4806 | Yes |
| 39 | A2M |  |  | 184 | 0.309 | 0.4808 | Yes |
| 40 | SPTBN1 |  |  | 194 | 0.300 | 0.4796 | Yes |
| 41 | CAST |  |  | 202 | 0.294 | 0.4804 | Yes |
| 42 | DKK3 |  |  | 205 | 0.293 | 0.4870 | Yes |
| 43 | ACSL3 |  |  | 207 | 0.291 | 0.4947 | Yes |
| 44 | GNG11 |  |  | 210 | 0.289 | 0.5012 | Yes |
| 45 | GNG12 |  |  | 212 | 0.287 | 0.5088 | Yes |
| 46 | FERMT2 |  |  | 219 | 0.283 | 0.5105 | Yes |
| 47 | DAG1 |  |  | 228 | 0.279 | 0.5098 | Yes |
| 48 | PRRX1 |  |  | 229 | 0.278 | 0.5182 | Yes |
| 49 | MEG8 |  |  | 230 | 0.278 | 0.5267 | Yes |
| 50 | CPM |  |  | 236 | 0.271 | 0.5291 | Yes |
| 51 | EHD2 |  |  | 240 | 0.267 | 0.5338 | Yes |
| 52 | LEPROT |  |  | 259 | 0.259 | 0.5210 | Yes |
| 53 | ATP1B2 |  |  | 260 | 0.258 | 0.5288 | Yes |
| 54 | WWTR1 |  |  | 263 | 0.256 | 0.5343 | Yes |
| 55 | ZFP36L1 |  |  | 287 | -0.255 | 0.5156 | No |
| 56 | CPE |  |  | 315 | -0.262 | 0.4925 | No |
| 57 | SLK |  |  | 343 | -0.266 | 0.4696 | No |
| 58 | ADA |  |  | 371 | -0.273 | 0.4469 | No |
| 59 | C11orf96 |  |  | 372 | -0.274 | 0.4552 | No |
| 60 | APOLD1 |  |  | 374 | -0.275 | 0.4624 | No |
| 61 | SLC12A2 |  |  | 389 | -0.279 | 0.4548 | No |
| 62 | CTBP2 |  |  | 393 | -0.280 | 0.4598 | No |
| 63 | TXNIP |  |  | 404 | -0.284 | 0.4569 | No |
| 64 | CHRAC1 |  |  | 435 | -0.294 | 0.4314 | No |
| 65 | BCHE |  |  | 471 | -0.305 | 0.4004 | No |
| 66 | PLEKHH2 |  |  | 473 | -0.307 | 0.4086 | No |
| 67 | EPB41L2 |  |  | 488 | -0.310 | 0.4019 | No |
| 68 | RBPMS |  |  | 519 | -0.320 | 0.3772 | No |
| 69 | DOCK10 |  |  | 520 | -0.320 | 0.3869 | No |
| 70 | SESN3 |  |  | 526 | -0.322 | 0.3909 | No |
| 71 | JMJD1C |  |  | 597 | -0.356 | 0.3213 | No |
| 72 | C18orf54 |  |  | 619 | -0.366 | 0.3082 | No |
| 73 | PID1 |  |  | 645 | -0.385 | 0.2912 | No |
| 74 | ZEB1 |  |  | 653 | -0.396 | 0.2952 | No |
| 75 | EGR1 |  |  | 656 | -0.398 | 0.3050 | No |
| 76 | SLC25A5 |  |  | 695 | -0.426 | 0.2743 | No |
| 77 | MAP3K1 |  |  | 702 | -0.431 | 0.2805 | No |
Table: GSEA details [plain text format]

  

Fig 2: RICHARDS\_NOWAKOWSKI\_SCIENCE\_2017\_MURAL\_UPREG: Random ES distribution      
 Gene set null distribution of ES for **RICHARDS\_NOWAKOWSKI\_SCIENCE\_2017\_MURAL\_UPREG**
